# *In Vitro* Mean Red Blood Cell Volume Change Induced by Diode Pump Solid State Low-Level Laser of 405 nm

**DOI:** 10.1089/pho.2015.4043

**Published:** 2016-05-01

**Authors:** Mustafa S. Al Musawi, Mohamad Suhaimi Jafar, Bassam T. Al-Gailani, Naser Mahmoud Ahmed, Fatanah Mohamad Suhaimi, Nursakinah Suardi

**Affiliations:** ^1^ School of Physics, Department of Medical Physics, Universiti Sains Malaysia, Pulau Pinang, Malaysia.; ^2^ Department of Physiology, College of Medicine, Al-Mustansiriya, Iraq.; ^3^ Advanced Medical and Dental Institute, Universiti Sains Malaysia, Bertam Kepala Batas, Pulua Pinang, Malaysia.

## Abstract

***Objective:*** This study was conducted to investigate the effects of low-level laser (LLL) doses on human red blood cell volume. The effects of exposure to a diode pump solid state (DPSS) (*λ* = 405 nm) laser were observed. ***Background data:*** The response of human blood to LLL irradiation gives important information about the mechanism of interaction of laser light with living organisms. ***Materials and methods*** Blood samples were collected into ethylenediaminetetraacetic acid (EDTA)-containing tubes, and each sample was divided into two equal aliquots, one to serve as control and the other for irradiation. The aliquot was subjected to laser irradiation for 20, 30, 40, or 50 min at a fixed power density of 0.03 W/cm^2^. Mean cell volume (MCV) and red blood cell (RBC) counts were measured immediately after irradiation using a computerized hemtoanalyzer. ***Results:*** Significant decrease in RBC volume (*p* < 0.05, *p* < 0.0001, *p* < 0.0001, and *p* < 0.05, respectively) was induced with variation in laser doses.The highest response was observed with an exposure time of 40 min. This result was reproduced in RBCs suspended in a buffered NaCl solution. In contrast to this finding, laser-induced RBC volume change was completely abolished by suspending RBCs in a solution containing a higher concentration of EDTA. ***Conclusions:*** It was suggested that LLL can reduce RBC volume possibly because of the increased free intracellular Ca^+2^ concentrations, which activate Ca^+2^-dependent K^+^ channels with consequent K^+^ ion efflux and cell shrinkage.

## Introduction

Diode pump solid state (DPSS) lasers are widely used for low-level laser light therapy (LLLT). DPSS laser light is monochromatic, confining all the beam energy into a narrow wavelength band, which can be important for biomedical, holographic, spectroscopic, and other applications. The advantages of DPSS laser are its compactness, efficiency, and cost effectiveness in comparison with other types of lasers.^1–3^

Over the last few decades, many studies have been performed to investigate effects of LLL irradiation in medical science.^4,5^ However, the mechanisms of its effect on human blood components still have not been discovered sufficiently, and it is still a topic for discussion. The response of human blood to LLL irradiation gives important information about the interaction mechanism of laser irradiation with a living organism.^4,6^ However, some studies have examined the effects of LLL irradiation on human blood, especially on the red blood cells (RBCs).^6–10^ This study has been conducted to further understand the response of RBCs to LLL irradiation. However, the information regarding the response of human blood parameters is still lacking, such as RBC volume to low laser light irradiation.

Blood properties, of patients with different pathologies, are modified with LLL irradiation.^11^ Laser irradiation improves microcirculation, and modulates rheological properties of blood pathology.^12^ Laser irradiation induces conformational transitions of the red blood cell membrane, which is related to changes in the structural states of both erythrocyte membrane proteins and lipid bilayer, resulting in changes in the activity of membrane ion pumps.^13^ The laser-induced changes in various biological objects, such as blood components, have been the focus of previous studies.^14^ It is well known that the cell membrane is the first line of interaction between cellular machinery and the outer environment. It is the site of key events in laser interaction with cells.^5^ The red blood cell membrane is most prominent because of its simplicity, availability, and physiological importance.^15^ A number of studies have been conducted, both *in vitro* and *in vivo*, that shows the significant influence of laser irradiation on the red blood cell functions. Moreover, a group of researchers found undetectable effects of laser exposure.^13^

This study aimed to investigate the *in vitro* effects of different low-level DPSS laser doses of the wavelength 405 nm on mean cell volume (MCV) in human blood.

## Materials and Methods

### Blood samples

Fresh human blood samples were collected at the Hematology Laboratory, Advanced Medical and Dental Institute (AMDI), Universiti Sains Malaysia (USM), Pinang, Malaysia. Apparently healthy adults were selected and convinced to participate in this study after being briefed about the aims and objectives of the study and assured about the privacy of the confidential data. The blood samples collection was approved by USM Research Ethics Committee (Human). The blood samples, of 5 mL, were collected from all the participants through a venipuncture into ethylenediaminetetraacetic acid (EDTA)-containing tubes (1.3 mg/mL of blood) as anticoagulant. The samples were then processed immediately after collection. Each sample was divided into two equal parts to be used as an unirradiated control sample and an irradiated sample. The samples were analyzed within 2 days after collection.

### Laser irradiation

The blood samples were irradiated with a low-power laser beam of 405 nm from a DPSS (model F Series, Changchun Dragon Lasers Co.) (Transverse mode, TEM_00_), with an output power of 10 mW. Optical power was calibrated using a Newport multifunction optical meter (model 2936-C). The blood samples, contained in 2.5 mL tubes, were irradiated with a laser beam of 0.332 cm^2^, with a power density of 0.03 W/cm^2^, for 20, 30, 40, and 50 min. The delivered dose, for each irradiated group, was 36, 54, 72, and 90 J/cm^2^, respectively. The laser beam was directed normally to the blood-sample-containing tubes, from up to down (only one point, in the center of the test tube). The irradiation was performed at room temperature (23 ± 2°C).

### MCV measurement

The MCV was measured before and after irradiation for each blood sample using a computerized hematology analyzer (Mindray BC-3200). All the measurements were performed immediately after irradiation for the irradiated samples. Data for the MCV and RBC count are an average of four repeated measurements.

### Processing and irradiation of RBC suspension

Separation of blood components was achieved by centrifugation of blood, using bench centrifuge (MP 6000 R, Eltek. Co. India) at 3000 rpm for 5 min. The plasma, buffy coat, and uppermost layers of packed RBCs were discarded. The packed RBCs were washed thrice in 0.9% NaCl solution through resuspension and recentrifugation of the RBC suspension. Three aliquots of 250 μL of washed packed RBCs were dispensed into three tubes. One of the tubes served as a control in which the red blood cells were resuspended in a 250 μL solution containing in *m*M: NaCl 150, Tris base 10, and pH 7.4. The washed RBCs were resuspended in a second tube, in the same solution as was contained in tube one. The contents of this tube were exposed to 72 J/cm^2^ of DPSS laser light for 40 min. In the third tube, the washed RBCs were resuspended in a 250 μL solution containing in *m*M: NaCl 150, Tris base 10, EDTA 10, and pH 7.4. The contents of this tube were also exposed to laser radiation exactly as in the second tube. The pre-irradiation MCV and post-irradiation MCV of the RBCs of the suspended samples in each blood sample were measured using a computerized hematology analyzer.

### Statistical analysis

Statistical analysis and calculation were performed using SPSS software. The difference between the controlled and irradiated samples was evaluated by applying a paired sample student *t* test. The *p* value was determined according to analysis of the significance of difference. A *p* value of <0.05 was considered significant. All results are expressed as mean *±* SD.

## Results

### Effects of 405 nm laser irradiation on RBC volume

The blood samples were irradiated, *in vitro,* using a 405 nm DPSS laser light with 10 mW irradiation power. No hemolysis or morphological changes of the erythrocytes were observed. No significant differences in RBC count were observed between pre-irradiated and post-irradiated samples, as shown in Table 1.

**Table 1.** RBC Count and MCV Before and After LLL Irradiation

*Dose (J/cm^2^)*	*Blood parameters*	*Unirradiated mean ± SD*	*Irradiated mean ± SD*	*Difference mean ± SD*	p *Value*
36	RBC(×10^12^)/L	4.90 ± 0.36	4.90 ± 0.36	0.0005 ± 0.007	0.683
	MCV (fL)	89.11 ± 2.58	88.92 ± 2.57	0.185 ± 0.49	0.049
54	RBC(×10^12^)/L	4.66 ± 0.54	4.66 ± 0.55	0.001 ± 0.01	0.527
	MCV (fL)	88.75 ± 2.04	88.39 ± 2.13	0.352 ± 0.45	0.0001
72	RBC(×10^12^)/L	5.18 ± 0.48	5.17 ± 0.48	0.004 ± 0.02	0.386
	MCV (fL)	89.82 ± 2.99	89.42 ± 3.07	0.396 ± 0.235	0.0001
90	RBC(×10^12^)/L	5.23 ± 0.50	5.23 ± 0.50	0.0002 ± 0.003	0.697
	MCV (fL)	89.09 ± 2.49	89.04 ± 2.51	0.049 ± 0.120	0.033

RBC, red blood cell; MCV, mean cell volume; LLL, low-level laser.

**FIG. 1.**
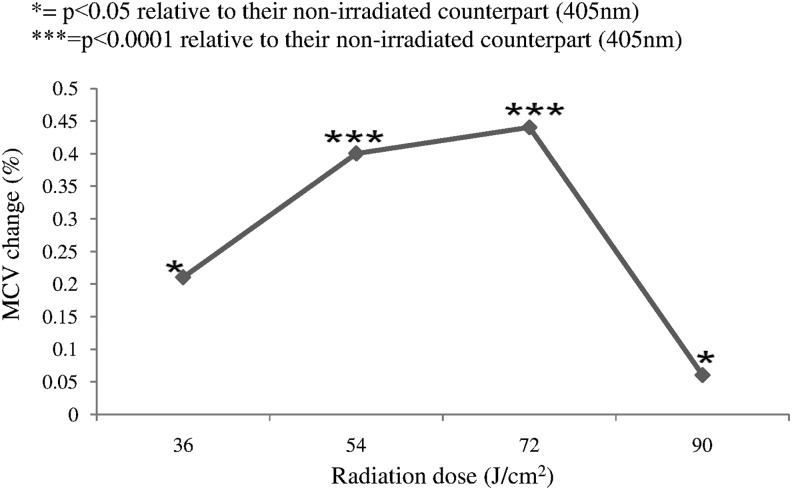
Percent change in mean cell volume (MCV) with change in radiation dose (*n* = 30 for each specific dose). *p < 0.05 relative to their unirradiated counterpart (405 nm). *p < 0.0001 relative to their unirradiated counterpart (405 nm).

The MCV change is observed as a function of irradiation dose as summarized in Fig. 1. Various doses were applied and found able to induce significant changes in RBC volume (*p* < 0.05, *p* < 0.0001, *p* < 0.0001, and *p* < 0.05, respectively). It is clear that *in vitro* irradiation of human blood to DPSS 405 nm laser significantly decreases the MCV at doses of 36, 54, 72, and 90 J/cm^2^. The dose of 72 J/cm^2^ is optimum; it results in maximum MCV decrease, 0.44%, of healthy human RBCs, in comparison with unirradiated RBCs.

### Effects of 405 nm laser irradiation on RBCs suspended in artificial suspending media

**FIG. 2.**
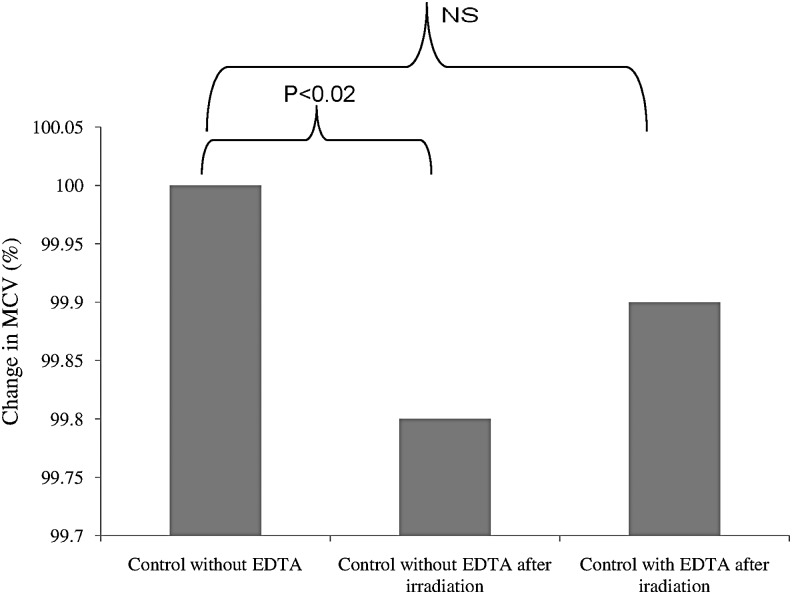
Post-irradiation mean cell volume (MCV) of red blood cells (RBCs) in the absence and presence of ethylenediaminetetraacetic acid (EDTA) (number of samples = 14).

The MCV of RBCs suspended in EDTA-free solution, irradiated to 72 J/cm^2^ from a 405 nm laser, is significantly smaller (by 0.2%) than that of unirradiated RBCs suspended in same solution, as shown in Fig. 2. This clearly indicates that irradiation causes a significant decrease in RBC volume. In contrast to this finding, laser-induced RBC volume change was found to be completely abolished by suspending RBCs in EDTA-containing solution.

## Discussion

This study was aimed to determine the effectiveness of the 405 nm DPSS laser as a LLL on the RBC volume in human blood. The laser radiation in the violet spectral region has been used in this study because hemoglobin absorbs maximum light in this region as compared with other spectral regions, this result in light penetration deep into living tissue.^16^ The LLL is a special type of laser that affects biological systems through nonthermal means. In this system, temperature elevation in the irradiated tissue is limited to <0.1–0.5°C.^16,17^ In this study, the RBCs were exposed to different LLL doses and significant decrease in the MCV is observed. Similar results are reported previously where LLL was used with different wavelengths.^18,19^ Four different doses were selected to perform intercomparisons between the doses. The comparison was made with an unirradiated control group. The maximum decrease in MCV was observed with a dose of 72 J/cm^2^. This LLL dose produces optimum results as compared with any other dose. This illustrates the basic concept of the biphasic dose response or hormesis.^20^ Sufficient energy is needed to overcome the threshold to obtain a response; however, energy greater than threshold energy is required to replace biostimulation with bioinhibition.^21,22^

The MCV of RBCs suspended in EDTA-free solution and irradiated with dose of 72 J/cm^2^ at 405 nm is shown in Fig. 2. A reduction in RBC volume could be explained on the basis of ion fluxes. It is possible that the reduction of RBC volume upon irradiation (while the cells are suspended in EDTA-free solution) is caused by the activation of Ca^+2^-dependent K^+^channels. Under normal conditions, it is possible to expect that free Ca^+2^ is present at a very low concentration (micromolar or submicromolar level) in the solution used throughout this study. This is because of the use of distilled water rather than deionized water for preparation of solutions. Low-intensity laser induced conformational transitions of the RBC membrane, which are related to the changes in the activity of the membrane ion pumps and, as a result, changes in membrane ion flows.^13^

It is possible to suggest that laser light has the ability to disrupt the cell membrane layers. This disruption may lead to open pores for Ca^+2^ ions. The free Ca^+2^ then passes from the extracellular solution (EDTA-free solution) to the intracellular RBC fluid through cell membrane pores induced by laser light. The increased free intracellular Ca^+2^ concentrations stimulate the activation of Ca^+2^-dependent K^+^ channels. Upon stimulation of such channels, K^+^ passes from inside of the cells, where the concentration of K^+^ is high, to the extracellular fluid, where there are no K+ ions (K^+^ efflux according to concentration gradient). This K^+^ movement is also associated with water molecules movement (law of osmosis). Accordingly, smaller RBCs were yielded. Adding EDTA to the suspending solution was shown to completely obliterate the effect of Ca^+2^ in inducing such volume changes upon irradiation by chelating all available free extracellular Ca^+2^. The results may also explain the reduction of RBC volume upon laser irradiation in whole blood samples collected in EDTA-containing tubes. This is because the amount of EDTA in such tubes is enough to chelate Ca^+2^ and prevent blood coagulation cascade, but still not high enough to chelate all available free Ca^+2^ ions.

## Conclusions

The maximum decrease in MCV is observed with the irradiation dose of 72 J/cm^2^. Accordingly, it is possible to conclude that this is the optimum dose that produces optimized results. The exposure of RBCs to LLL can reduce RBC volume possibly because of the increased free intracellular Ca^+2^ concentrations, which activate Ca^+2^-dependent K^+^ channels.
